# Dynamics of Psychological Crisis Experience With Psychological Consulting by Gestalt Therapy Methods

**DOI:** 10.5539/gjhs.v7n4p130

**Published:** 2014-12-31

**Authors:** Liliya Raifovna Fahrutdinova, Dzhamilia Renatovna Nugmanova

**Affiliations:** 1 Kazan (Volga Region) Federal University, Kremlyovskaya Street, 18, Republic of Tatarstan, 420008, Kazan, Russian Federation

**Keywords:** experience, corporeal, emotional, cognitive elements, psychological consulting, crisis situations

## Abstract

Dynamics of experience as such and its corporeal, emotional and cognitive elements in the situation of psychological consulting provisioning is covered. The aim of research was to study psychological crisis experience dynamics in the situation when psychological consulting by gestalt therapy methods is provided. Theoretical analysis of the problem of crisis situations, phenomenon and structural, and dynamic organization of experience of the subject of consulting have been carried out. To fulfill research project test subjects experience crisis situation have been selected, studied in the situation when they provided psychological consulting by methods of gestalt therapy, and methodology of study of crisis situations experience has been prepared. Specifics of psychological crisis experience have been revealed and its elements in different stages of psychological consulting by gestalt therapy methods. Dynamics of experience of psychological crisis and its structural elements have been revealed and reliable changes in it have been revealed. Dynamics of psychological crisis experience and its structural elements have been revealed and reliable changes in it have been revealed. “Desiccation” of experience is being observed, releasing its substantiality of negative impression to the end of consulting and development of the new experience of control over crisis situation. Interrelations of structural elements of experience in the process of psychological consulting have been shown. Effecting one structure causes reliable changes in all others structural elements of experience. Giving actual psychological help to clients in crisis situation by methods of gestalt therapy is possible as it was shown in psychological consulting sessions. Structure of client’s request has been revealed – problems of personal sense are fixed as the most frequent cause of clients’ applications, as well as absence of choices, obtrusiveness of negative thoughts, tend to getting stuck on events took place in the past, drawing into oneself, etc.

## 1. Introduction

The present research covers experience dynamics with consulting. The problem of experience of crisis situations becomes the most topical problem of clients appealing to psychologist. In this regard analysis of the process of crisis situations experience of a person, improvement of technical tools in the work with crisis experience, development of skills that allow practitioner to reveal client’s problem as critical promptly, practitioner’s resistance and the qualities of presence with a client, mastering of general strategy and tactics of work with different types of crisis are important for psychologist going in for psychological consulting (Johnson, 2003).

Psychological literature view psychological crisis in most cases as acute emotional state developing in the situation of blocking purposeful life activity of a man (Olatunji, Wolitzky-Taylor, Sawchuk, & Ciesielski, 2010); as discreet moment of personality development in situation of potential or real threat to satisfying of fundamental necessities; as problem that a man cannot go away of and that he cannot solve in short time and by the way he is used to (Bonanno, Pat-Horenczyk, & Noll, 2011). Most researchers distinguish development crisis, crisis of loss and trauma crisis related to the situations of violence, suicide, severe disease of a client appealing for aid or his relatives, etc. (Golan, 1978; Erikson, 1963). Despite significant difference in the sources of crisis, certain situations and the content of experiences of clients the most general phenomenological manifestations of crisis may be distinguished (Paul Rees & Niels Seaton, 2011). These are the feeling of hopelessness, apathy, disappointment, depression, tiredness, loneliness, oppression, etc. (Karvasarski, 1998.), general high affective tension (Starshenbaum, 2005).

Dealing with experiences is the main psychological technologies of consulting activity of psychological practitioner. Research of internal dimension of the process of psychological consulting actualized via experience has been carried out for the first time. According to L.S. Vygotskii internal dimension of social situation is being represented via experience (Vygotskii, 2000). Everything that goes on during consultation may be researched in no way but via experience of a man. According to Dilthey psychological “research grows from experience itself and should constantly preserve solid roots in it to be healthy and grow” (Dilthey, 2002). Definition and its operationalization are represented in Fakhrutdinova’s concept of structural and dynamic organization of experience (Fakhrutdinova, 2012). Corporeal, emotional and cognitive elements of experience have been analyzed in this work as well as characteristic of experience as a whole. So we relied on operational definition of experience as a total of corporeal self-sentiment, emotional and cognitive processes, united by the common object (Fakhrutdinova, 2009)

Object of the research: experience of crisis situation.

Subject of research: psychological consulting effect on structural organization of crisis situation experience.

The following research hypothesis had been made: the structure of crisis situation experience is reliably changing and its intensity decreases in the process of psychological consulting.

The aim of research was to study subject’s experience dynamics in the situation of psychological consulting provisioning.

The following tasks have been solved in the research:

1) Research of corporeal, emotional and cognitive elements of psychological crisis experience in the situation of psychological consulting by gestalt therapy methods.
2) Analysis of the structure of client’s appeal for psychological consulting.
3) Theoretical analysis of the problem of psychological consulting of clients in situation of crisis experience.
4) Preparation of research project including selection of test subjects experience crisis situation, survey of test subjects in the situation of psychological consulting by gestalt therapy methods, preparation of methodology of crisis situations experience study.

## 2. Method

We used questionnaire “Experience CEC” that includes the following variables to study experience.

The scale “Experience” measures modality (negative and positive character) and intensity of experience (Fakhrutdinova, 2012). Conception of corporeality is represented by the following operational concept: corporeality is a total of corporeal self-sentiment of a man represented on exteroreceptors’, interoreceptors’ and proprioceptors’ level (Fakhrutdinova, 2012). Scale “Corporeality” reflects modality and intensity of corporeal manifestations of experience (Fakhrutdinova, 2012). The scale “Corporeal” reflects the volume of corporeal dimension, the scale “Emotional” reflects of volume of emotional dimension, the scale “Cognitive” - the volume of cognitive dimension (thoughts, images associations and others) in substantivity of experience (Fakhrutdinova, 2009).

Gathered data have been subjected to qualitative and quantitative analysis. Method of statistic checking based of Student’s distribution was used for data processing.

Psychological consulting of persons in crisis situations (some test subjects have been undergoing midlife crisis, some suffered from relationship problem, loss of relatives, severe diseases, firing, etc.) in the period of acute experience without the capability of control situation have been provided in the course of research. Test subjects’ survey was made by questionnaire “Experience CEC” in the course of psychological consulting: in the beginning of consulting, after 20-30 minutes after and at the end on 50-60th minute of a session. Analysis of the cause of appeal was conducted during consulting by the way of conversation.

30 test subjects had been selected from clients applied for psychological aid in the age of 20-44, 4 men, 26 women. Age of most test subjects was from 35 to 44 that correspond to the period of midlife crisis. Other appeals had situational character, i.e. depended on situation in a persons’ life.

## 3. Results

**Figure 1:**
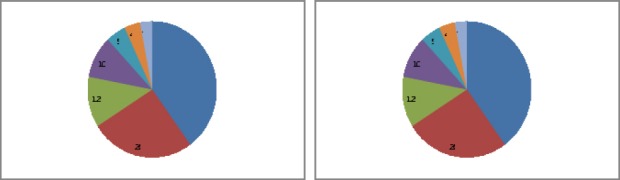
Structure of client’s appeal

In our research test subjects have noted for the period of appeal for psychological aid that corporeal experience share was 50%-60% of the space of their experience and evaluate the strength of their experience as very severe negative experience (energy component of experience). Sticking on negative images relating to the past and future was observed as time dimension. The picture of dissociation of body and consciousness was observed as spacial dimension. Bodily symptoms – trembling hands, tiredness of separate bodyparts or specifics of perception of a certain situation – the light is fading, sensitivity is being lost of on the contrary is sharp were often observed in the structure of clients’ appeals. The following causes of appeal were fixed: loss of personality sense (40% respondents), personality senses conflict (26%), lack of choice (12%), obtrusiveness of negative thoughts (10%), sticking to events of the past (5%), drawing into themselves (4%) and other factors (3%) (see Figure 1).

Loss of personality sense us marked by blue color, personality senses conflict – by brown, lack of choice – green, obtrusiveness of negative thoughts – violet, sticking to events of the past – light blue, drawing into themselves – orange and grey – other factors.

**Figure 2:**
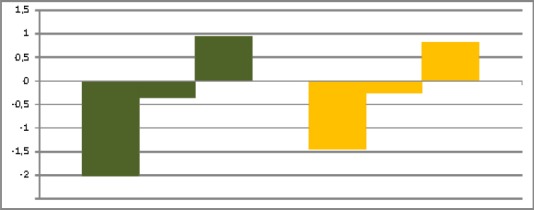
Dynamics of scales “Experience” and “Corporeality” in the beginning, middle and the end of psychological consulting

Figure 2 shows changing of scales “Experience” and “Corporeality” of questionnaire “Experience CEC” in the process of psychological consulting. Statistical check method based on the Student’s distribution allows proving reliability of changing data (p=0.01). It was shown that changing of experience characteristics as a whole and its corporeal element had been reliably changing in the process of consulting. Reliable changes of indicators of experience between the beginning and the middle of consulting, between the middle and the end in the situation of psychological consulting provisioning have been observed.

Scale “Experience” is marked by green color, “Corporeality” - by yellow; in each group of color first column corresponds to beginning, second – middle and third – to the end of psychological consulting. Vertical axis corresponds to intensity of experience from -3 to 3 points with negative values corresponding to negatively colored experience and positive values – to positive experience.

**Figure 3:**
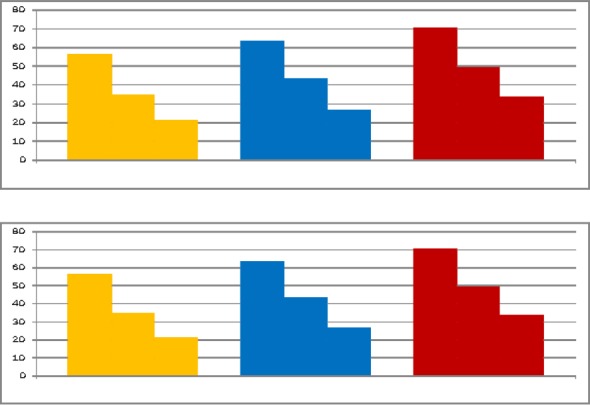
Changes of corporeal, emotional and cognitive elements of experience in the beginning, middle and the end of psychological consulting

Figure 3 reflects reliable changes of corporeal, emotional and cognitive elements of experience in the beginning, middle and the end of psychological consulting.

## 4. Discussion

Analysis of clients’ appeal has shown that major share of problems of test subjects are related to sense structures of consciousness that is confirmed by researches of F.E. Vasiliuk, L.R. Fakhrutdinova (Fakhrutdinova, 2008, 2009). F. E. Vasiliuk views experience as psychic activity of sense production in critical situations (Vasiliuk, 1984).

Scale “Corporeal” is marked by yellow, “Cognitive” – by blue, “Emotional” – by red color; first column of each color group corresponds to beginning, second – middle and third – to the end of consulting. Vertical axis corresponds to intensity of experience in percent from the highest – 100 and lowest – 0 that corresponds to calm state.

In the course of consulting in first 25-30 minutes we worked mainly with corporeal self-sentiments of a client on the base of psychological method SIBAM Mazur E.S., 2011), (Peter A. Levin, 1997). According to Fakhrutdinova’s concept of experience in the first stage of experience corporeal element dominates so work with corporeal dimension of experience allows find necessary resources to show, live through and release impressions that cause negatively colored experience and consequently for creating new experience of control (Fakhrutdinova, 2008).

As Figure 2 shows modality of experience and corporeal element are changing in the course of consulting from “minus” to “plus” i.e. from evaluation of crisis situation as severe, negative to positive, comfort. This fact shows that the process of impression processing had started in the process of reflection on experience of crisis situation. As we have shown in our previous researches the sign of experience changes from negative to positive in the process of releasing substantivity of experience from impression that causes negatively colored experience in the process of reflection on experience of impression (Fakhrutdinova, 2008). Correlation of research results allows making conclusion that in the process of consulting processes of reflection had been started, critical situation had been perceived that led to the process of desiccation of experience and gradually releasing it from negative impression of crisis situation. The process of “desiccation” of experience, lowering of its’ intensity in all elements is shown in Figure 3. According to F. Perls a man has a system of contacts in each moment (The Self) (Perls, Goodman, & Hefferline, 1951). This system of contacts creates impression and feeds experience. In the course of consulting meeting it becomes possible to develop the process of experience in the present moment. The process goes on due to recognition, increase and concentration until tension is not run out (Polster & Polster, 1973). In gestalt therapy the awareness that “it is what I think, perceive, feel and do” comes up to development of sensation (Robin, 2004). The situation of overcoming crisis ends up with development of sense – the outcome of this living through by individual creative adaptation to situation (Hollis, 2008).

## 5. Conclusion

We have revealed patterns of experience dynamics in the process of psychological consulting by gestalt therapy methods that may be presented in the following resume:

1). Loss of personality sense (40% respondents), personality senses conflict (26%), lack of choice (12%), obtrusiveness of negative thoughts (10%), sticking to events of the past (5%), drawing into themselves (4%) and other factors (3%) are the reasons of clients; appeals. Giving actual psychological help to clients in crisis situation by methods of gestalt therapy is possible as it was reliably shown in psychological consulting sessions.
2). Dynamics of psychological crisis experience and its structural elements have been revealed and reliable changes in it have been proved. Dynamics of experience of psychological crisis and its structural elements have been revealed and reliable changes in it have been proved. Interrelations of structural elements of experience in the process of psychological consulting have been shown: effecting one structure causes reliable changes in all others structural elements of experience. “Desiccation” of experience is being observed, freeing its substantiality of negative impression to the end of consulting and development of the new experience of control over crisis situation.

Further research of experience dynamics of psychological crisis in the situation of psychological consulting provisioning but gestalt therapy methods may be related to the study of spacial and temporal, information and energy, psychosemantic and other characteristics of experience. Analysis of consulting session with this appeal and research of experience dynamics on the first several meetings with consultant may be interesting. Research of experience dynamics in the situation of psychological consulting provisioning by methods of other psychotherapeutic schools (psychoanalysis, existential, psychodrama and others) may be promising.
